# Isolated renal hydatid disease: laparoscopic approach to an uncommon entity (case report)

**DOI:** 10.11604/pamj.2021.38.175.27993

**Published:** 2021-02-16

**Authors:** Saket Kumar, Saurabh Singla, Ausaf Ahmad, Nishant Kurian, Venkat Rao Chidipotu

**Affiliations:** 1 Department of Surgical Gastroenterology, Indira Gandhi Institute of Medical Sciences, Patna, Bihar, India

**Keywords:** Renal hydatid cyst, laparoscopic, isolated, echinococcal cyst, case report

## Abstract

Hydatidosis is a parasitic disease, endemic in various parts of the World. It frequently involves liver and lungs and, rarely, other organs as well. Isolated renal hydatidosis is a rare entity that accounts for less than 3% of all hydatid cases. Surgery remains the mainstay of treatment. We hereby report a case of isolated renal hydatid cyst involving left kidney that was managed by laparoscopic approach.

## Introduction

Human hydatidosis is endemic in several parts of World including Indian subcontinent, parts of Africa and Middle-East Asia. The disease usually occurs in persons having close contact with dogs, sheep and cattle [1]. In humans, hydatid disease is usually found in liver (75%) and lungs (15%), while 10% are found in rest of the body. Genitourinary involvement occurs in 2-4% of cases, and kidney is most commonly affected organ. Renal hydatidosis may occur when larvae reach the organ through the bloodstream, lymphatics or direct invasion. Kidney involvement is usually the manifestation of disseminated disease and isolated renal hydatidosis is very rare [1]. We present a case of young male who presented to us with isolated left renal hydatid cyst and was managed laparoscopically. We have obtained written consent from the patient for reporting his medical condition in the medical literature. The report has been prepared according to the CARE guideline.

## Patient and observation

**Figure 1:**
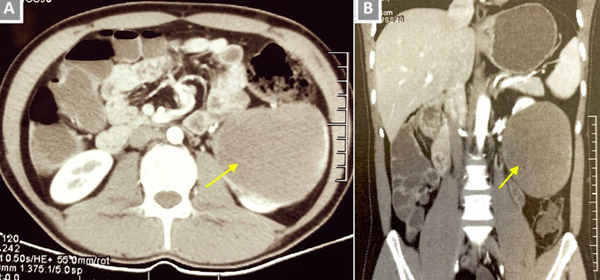
A) axial view CT scan of the abdomen showing a well-defined complex cystic lesion, arising from the inferio-medial aspect of the left kidney (arrow); B) coronal view

A 26 years old gentleman, presented to outpatient department with the complaints of pain in left hypochondrium for the last 5 to 6 months. The pain was initially mild, intermittent and dull in nature. However, since last one month the pain was constant, requiring daily oral analgesics. There was no history of fever, anorexia, weight loss or any urinary or bowel symptoms. There was no history of any abdominal trauma as well. He was non-vegetarian, teetotaler and did not smoke. On inquiry he revealed past contact with pet dogs till 5 years ago. On examination, his vitals were within normal limits. Abdominal palpation revealed a vague, non-tender lump in the left hypochondrium. The lump was immobile and there was no movement with the respiration either. Blood tests and urinalysis were normal. Patient was also evaluated elsewhere before reporting to us. Ultrasound abdomen done there showed a well-outlined complex cystic lesion, measuring 11 x 8cm containing echogenic fluid collection and arising from infero-medial aspect of left kidney. Computerized tomography (CT) scan reported similar findings (Figure 1).

**Figure 2:**
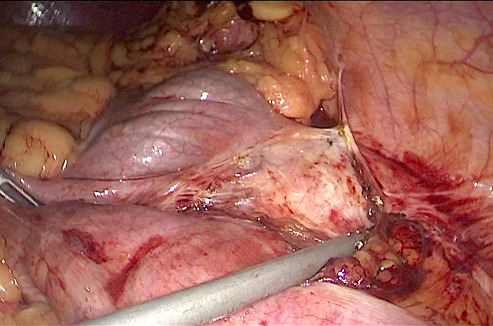
intraoperative image showing the exposure of hydatid cyst after mobilization of the descending colon

**Figure 3:**
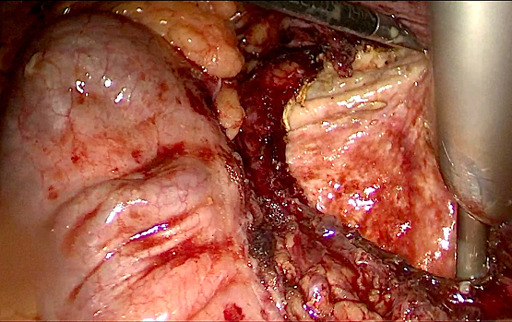
laparoscopic renal hydatid cyst de-roofing was done and daughter cysts were evacuated

Once the diagnosis of renal hydatid cyst was made, patient was started on Albendazole therapy at a dose of 400mg BD. He was planned for laparoscopic surgery after 2 weeks. The surgery was performed in supine position under general anaesthesia. Laparoscopic transperitoneal approach was used to access the cyst. Two 5-mm working ports were placed in the epigastrium and right paraumbilical region in the mid-clavicular line. Descending colon was reflected medially by incising the lateral attachment and cyst was exposed (Figure 2). Gauze piece soaked with hypertonic saline was placed around cyst to prevent the peritoneal dissemination. Cyst was then deroofed and contents were evacuated through a 10mm suction port in the left lumbar region. Precaution was taken to minimize the spillage. Cyst cavity was inspected at last for any residual daughter cyst and finally omentoplasty was done (Figure 3). Patient had an uneventful post-operative course and he was discharged on day 4 of surgery. He remains symptom-free at 1 year of follow-up.

## Discussion

Hydatid cystic disease is a parasitic infection caused by the larval form of Echinococcus species. The parasite inhabits the small intestines of infected dogs that serve as the primary host. The infected dogs excrete a large number of parasite eggs in its stool. Lamb, cattle, goats, or sometimes humans swallow these eggs. Humans are dead-end hosts and get infected through contact with the infected definitive host or ingesting contaminated soil, water or vegetables. After ingestion, the eggs hatch and produce larva in the small intestine of the host. The larvae penetrate the jejunum and gains access to the venous and lymphatic system. Through these routes, they reach various organs and develop into hydatid cysts. Liver and lungs act as primary and secondary filters, whereas genitourinary involvement is very rare [1].

Approximately 3% of the larvae that escape from these filters enter the systemic circulation and affect other systems. Kidneys are concomitantly affected as a part of secondary infection and isolated involvement is very rare [1, 2]. Most patients of renal hydatid are between 30 and 50 years of age [2]. The cyst is usually solitary and lies in the cortex of the kidney. Symptoms may vary depending on the size and extent of cyst. Patients with even a large cyst may remain asymptomatic. Symptomatic patients most commonly present with vague lumbar or flank pain, hematuria, and hypertension. The rupture of the cyst into the urinary system causes hydatiduria and is pathognomonic of renal hydatidosis. This finding though, is present only in 10-20% of the cases. A palpable mass is the most common clinical finding [2, 3].

**Figure 4:**
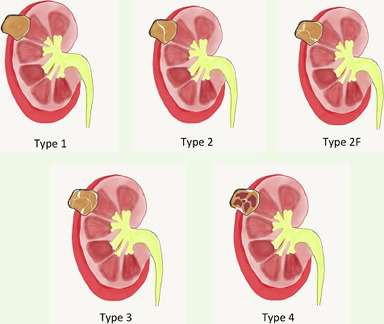
bosniak classification of renal cysts. Type 1: Thin wall, homogeneous, aseptate, no calcification; type 2: thin wall with fine calcifications and/or septations; type 2F: slightly thick and irregular wall, multiple thin septa with calcifications; type 3: uniform wall thickening with nodularity, multiple septa with calcifications and contrast enhancement; type 4: irregular soild-cystic lesion with prominent nodularity; solid enhancing components independent of septa

The differential diagnosis of renal hydatid cysts can be challenging even in endemic areas. The combination of clinical history, imaging studies and serological tests yields a reliable diagnosis in only 50% of cases [3]. Renal hydatid cyst may mimic renal cell carcinoma, renal abscess or complex benign cysts like renal lymphangioma, angiomyolipoma etc. [3, 4]. There are several reported cases where the renal hydatid was misdiagnosed as a malignant tumor and a radical surgery was performed [4-6]. Bosniak renal cyst type 2F and 3 often resemble renal neoplasm and cause diagnostic dilemma (Figure 4, Table 1). Serological tests are not very helpful in endemic areas, while eosinophilia is noted only in 50% of cases. Ultrasonography and CT scan are the most common imaging modality used to diagnose renal hydatid cyst. Multiloculated cyst containing daughter vesicles, floating membrane, and ring-like calcifications are diagnostic imaging findings [7].

**Table 1:** bosniak classification of renal cysts

Class	Description	Features on imaging	Malignant risk
1	Simple cyst	Anechoic, round, thin wall	<1%
2	Minimally complex	Single thin septation, thin calcification	<3%
2F	Minimally complex	Thin septation, thin calcification, hyperdense on CT	5-10%
3	Indeterminate	Thick or multiple septation, mural nodules	40-50%
4	Malignant cyst	Solid mass with cystic spaces	80-100%

The treatment modalities of renal hydatid cyst include medical management, percutaneous intervention and surgery by open or minimally invasive approach. Medical management in the form of Mebendazole or Albendazole has been the recommended for hepatic hydatid cyst [8]. However, because of low efficacy and associated adverse effects, the utility of medical treatment alone is limited. Medical treatment is used prophylactically in the perioperative period to avoid dissemination due to intraoperative spillage of cyst contents [8]. Our patient had received Albendazole treatment at a dose of 400 mg twice daily starting 2 weeks before and continued for 12 weeks after the surgery.

Percutaneous management of renal hydatidosis has also been described. PAIR (puncture, aspiration, injection of scolicidal agent, re-aspiration) can be an alternative treatment for renal hydatid cysts that do not communicate with the urinary collecting system [9]. Surgery remains the treatment of choice for renal hydatid cysts. Simple cyst deroofing or pericystectomy can manage superficial cysts with minimal involvement of the renal parenchyma. However, cases where the cyst involves the renal parenchyma, partial or total nephrectomy may be required. The surgery is aimed at the maximum preservation of the functional renal volume. Cyst excision with partial nephrectomy is possible in 75% of the cases. Total nephrectomy is rarely indicated and is reserved for cases where the kidney has been occupied entirely by the cyst and has become nonfunctional [10].

Recently, laparoscopic management of hydatid cyst has gained increasing popularity. Both transperitoneal and retroperitoneal laparoscopic approaches have been reported for renal hydatid cyst. The former approach has been considered superior in some studies as it allows more working space with less chances of cyst rupture [10]. Laparoscopic cyst deroofing with endocyst evacuation or laparoscopic cystopericystectomy are options for superficial renal cysts. Whereas laparoscopic transperitoneal partial as well as total nephrectomies have been described for management of hydatid cyst with deep renal extension [10]. The possible complications of laparoscopic cyst deroofing include risk of peritoneal dissemination, hemorrhage and urinoma or urinary fistula formation. The spillage of cyst fluid is known to produce anaphylactic reaction in some cases. Even after successful treatment of a hydatid cyst, there is still a risk for recurrence, however, its exact incidence in renal hydatidosis is not known due to rarity of this disease [8, 10]. Further studies are needed on such rare cases of primary renal hydatidosis to determine a universally accepted standard approach after comparing all available options.

## Conclusion

Surgery remains the mainstay of treatment of renal hydatid cyst. The growing evidence suggests that laparoscopic approach may be feasible and safe in treating this rare clinical entity.
